# The Teaching of Ethics and the Moral Competence of Medical and Nursing Students

**DOI:** 10.1007/s10728-020-00401-1

**Published:** 2020-09-17

**Authors:** Vera Sílvia Meireles Martins, Cristina Maria Nogueira Costa Santos, Patrícia Unger Raphael Bataglia, Ivone Maria Resende Figueiredo Duarte

**Affiliations:** 1 Centre for Research in Health Technologies and Information Systems (CINTESIS), University of Porto, R. Dr. Plácido da Costa, 4200-450 Porto, Portugal; 2 Department of Community Medicine, Information and Health Decision Sciences, Faculty of Medicine, University of Porto, Al. Prof. Hernâni Monteiro, 4200-319 Porto, Portugal; 3 Educational Psychology Department, Faculty of Philosophy and Science, São Paulo State University, Av. Hygino Muzzi Filho, 737- Bairro Mirante, Marília, São Paulo Brazil

**Keywords:** Ethics education, Teaching, Moral development, Medical students, Nursing students

## Abstract

In a time marked by the development of innovative treatments in healthcare and the need for health professionals to deal with resulting ethical dilemmas in clinical practice, this study was developed to determine the influence of the bioethics teaching on the moral competence of medical and nursing students. The authors conduct a longitudinal study using the Moral Competence Test extended version before and after attending the ethics curricular unit, in three nursing schools and three medical schools of Portugal. In this questionnaire the participant is confronted with three ethical dilemmas (related to theft, euthanasia and the torture of a terrorist) and asked to evaluate arguments for and against the attitude of the main character (Worker, doctor and judge). For both nursing and medical students, C-score was lower after the attendance of the ethics curricular units, with a statistically significant decrease in the total score (from 21 to 19.5 on average; *p* = 0.046) for nursing students and a decrease not statistically significant for medical students (from 23.2 to 22 on average; *p* = 0.358). A multivariate analysis did not find any association between this decrease and gender, course, or age. The phenomenon of moral segmentation was observed, with better performance in the worker and judge dilemma, than in the doctor dilemma. These results highlight the need to reflect on the curricular strategies that can be implemented for health professionals to better develop moral competence and decision-making, allowing for the provision of humanized health care.

## Introduction

In healthcare, innovative treatments are being developed, including new technologies that allow for improved treatment of diseases, which had been incurable and fatal. With an increased life expectancy and technological advances in medical care, health professionals must intervene in diverse situations. They are confronted daily with ethical dilemmas for which they must establish functional answers, as the life of the patient often depends on their response. The evolution in healthcare has led to a growing concern with the education of new health professionals and the curricular strategies implemented that allow the student and future professional to develop moral competence, safeguarding respect for the fundamental rights of those who are ill, thereby enabling them to improve their quality of life and dignity.

Kohlberg [10: 425] defines moral judgment competence as “the capacity to make decisions and to judge morally, based on internal principles, and to act in accordance with such judgments”. For the author, individuals go through an evolutionary sequence of universal stages, regardless of origin or cultural influences; however, not all of them reach optimal levels. Kohlberg presents six levels of development [11], grouped in three stages: the preconventional stage (levels 1 and 2), characteristic of most children, with individual judges varying in opinion based on their own interests, which includes fear about being punished. In the conventional stage (levels 3 and 4), present in most adolescents and adults, ‘correct’ moral action is based on social rules imposed by recognised authorities or institutions. The postconventional stage (levels 5 and 6) is only achieved by a minority of individuals, for whom action is based on universal moral principles, guided by reciprocity and equality. Kohlberg proposed that conditions that foster individual moral development, including intellectual, social, and educational awareness should be ensured [9]. According to Kohlberg, environments in which the individual develops the ability to work in groups, share decision-making, take responsibility for his/her actions, can also stimulate high levels of moral development.

Lind starts from Kohlberg’s definition of moral competence, citing that moral competence can be understood as “the ability to solve problems and conflicts on the basis of one’s moral principles through deliberation and discussion, instead of through violence, deceit, or bowing down to others” [14: 13]. The author bases this on Piaget’s Dual Aspect Theory to develop a new approach for the cognitive and affective aspects of moral behaviour. According to Lind [14], moral competence is not innate and does not develop on its own; it needs to be taught and reinforced. Lind developed a questionnaire, the Moral Competence Test (MCT), consisting of two ethical dilemmas, that allow the subject to show his competence in applying his moral structure in adverse situations, i.e., situations with which he does not share his opinion [4]. In the extended version of MCT the subject is confronted with three ethical dilemmas and asks for evaluative arguments for and against the attitude of the main character. In the first dilemma, this character is a worker who decides to break into his company to find evidence of management misconduct and then deliver this information to the authorities. In the second dilemma, a woman with terminal cancer asks the doctor to administer a dose of morphine to cause her death, which he accepts. In the third dilemma, a judge responsible for assessing a future terrorist attack is asked for authorisation to torture a woman who knows details of the attack, an action that the judge authorizes. According to Lind, the C-score which results from the application of the MCT, reflects the ability of the individual to choose arguments against or in favor of a moral option [14]. Thus, C-scores lower than 4.9 points indicate no or little moral competence, between 5 and 9.9 points some, but very low moral competence; between 10 and 19.9 points, less moral competence than seems to be need for choosing moral options in a dilemma situation; from 20 to 29.9 points, a moral competence sufficient to solve most ethical dilemmas; and above 30 points, a high moral competence [16].

Studies related to moral competence in medical and nursing students are still scarce. Lind, through a longitudinal study, found a marked decrease in the moral competence of medical students between the first and fifth semesters, and a more discrete decrease between the fifth and ninth semesters [12]. This regression was partially recovered between the ninth and thirteenth semesters. Schillinger, in a cross-sectional study, compared the moral competence scores of medical, psychology and business administration students, in three countries between the first and last year, and concluded that medical students show a decreased score of moral competence at the end of the course [22]. Slovacková and Slovácek, in a cross-sectional study, found that medical students had lower moral competence scores in the tenth semester compared to the second semester [23]. Hegasi and Wilson, in a cross-sectional study with medical students in the first and fifth years, found that students presented lower scores of moral competence in the fifth year [7]. Neves Feitosa et al. [18] found that medical students had lower moral competence scores in the eighth semester compared to the first semester.

Regarding nursing students, Buzgová and Sikorová observed that nursing students presented higher moral competence scores at the end of the course, compared to students in the first year [6]. On the other hand, Oliveira, while looking at the moral competence of nursing students, detected that in the ninth semester, students presented lower moral competence scores compared to students in the first semester [19]. The same author verified that the students presented very different scores of moral competence in relation to the worker and judge dilemma’s and the doctor’s dilemma [19]. In studies carried out by Bataglia et al. [3] in Brazil, and Moreno [17] in Mexico, they found disparate results for the two dilemmas of the MCT (Moral Competence Test) original version. Comparing the results in Brazil and Mexico with countries of the Europe and United States, very low scores were obtained for the doctor’s dilemma, related to euthanasia, and for the worker dilemma the results were equivalent to those in Europe, showing a lack of equivalence between the components of the test. Given these results, the judge dilemma was developed, in the same way related to the value of life, but the life of one person as opposed to the lives of many others. The scores obtained in judge dilemma are on average very close to the scores obtained in the worker dilemma [4]. According to Lind [15], when there are differences above five points between the doctor’s dilemma and remaining dilemmas, a phenomenon called moral segmentation occurs. Lind considers that segmentation means “the lowering of one’s moral competence in a dilemma situation in which a person turns over responsibility for judgment to an external authority like religion, ideology, military command, or professional ethics instead of using his or her own reason to solve the dilemma” [14: 184].This phenomenon could be related to certain institutions influence on decision-making, such as the case of religion as related to the value of life, regarding the doctor´s dilemma [4]. Also, according to Lind [15], changes of moral competence scores up to five points in the comparison of two groups or two moments of evaluation are not considered significant. The MCT is a questionnaire allowing for the evaluation of educational programmes, so that changes up to five points, comparing two moments of the questionnaire’s application that correspond to moral stagnation, suggest that educational programmes may not have significant impact on students’ moral competence, as found by several authors [18, 21, 22].

Analysing the ethics impact on moral competence development, several authors’ present factors towards improved moral development. Lind proposes a curricular and organisational revision of the medical course [12]; Schillinger refers to the importance of a good learning environment, which promotes role-taking and guided reflection [22]; Buzgová and Sikorová advocates changes in teaching methodology, with the introduction of methods that promote ethical argumentation [6]; Kim et al. [8] refers to the importance of follow-up by tutors, who are models of good practice in clinical teaching; Auvinen et al. [2] and Peter et al. [20] propose the discussion of ethical dilemmas as experienced in clinical teaching.

A study by Serodio, in which different methods of learning are applied to two groups of medical students, one group with traditional teaching methods versus the other group with the KMDD (Konstanz Method of Dilemma Discussion), found that in the traditional methods group, there was a decrease in moral competence scores, whereas with the KMDD, there was an increase, suggesting how different ethics teaching methods may promote students’ moral competence [21].

Results in these studies are not encouraging, as they apply to medical and nursing students, future health professionals, from whom moral action is expected to be adequate, indeed these professionals rely on norms and rules specific to their professions, which often take the form of deontological codes, and thus a professional morality, which allow them to make decisions in each specific situation they face [5].

This could indicate that teaching is not directed at promoting reflection or critical judgment, which are essential elements in decision-making of those who deal with ethical dilemmas in daily practice. Thus, we decided to investigate if the teaching of ethics can influence the moral competence of both medical and nursing students.

## Methods

### Study Design

A longitudinal, descriptive study was designed, which consisted of applying the Moral Competence Test extended version (MCTxt) questionnaire to nursing students from three nursing schools and medical students from three medical schools, located in the north and central regions of Portugal. The questionnaire was applied on two separate occasions, before and after attending the ethics curricular unit. This is taught in different years, as part of the first-year study programme in two nursing schools and the second-year programme in the remaining nursing school. In medical schools, the ethics unit is part of the first and fifth curricular year in one university and part of the third and fourth year in the others. All institutions, except for first year medicine, teach the ethics curricular unit in the second semester in which the application of the questionnaire was done. Each teacher agreed that the questionnaire would be applied at the beginning of the first and last lesson of this curricular unit, during the second semester of 2018.

The questionnaire was applied in the ethics classroom and was preceded by an explanation of the study’s objectives, with written consent requested from participants. The investigator remained to clarify doubts regarding completion of the questionnaire. Attached to it, participants were asked to provide the following information: gender, age and place of birth. The completion of the questionnaire was not restricted in time, as recommended by Lind [14], and it took about 15–30 min.

### Data Collection Instrument

The questionnaire was the MCTxt, composed of three moral dilemmas developed by Lind [13] and adapted to the Portuguese language by Bataglia et al. [3]. This instrument can simultaneously determine moral orientations (affective aspect) and moral competence (cognitive aspect) of moral action, as well as personal opinions. This questionnaire confronts the subject with three ethical dilemmas and asks for evaluative arguments for and against the attitude of the main character. Each dilemma has six arguments in favour and six arguments against, based on Kohlberg’s stages of moral development. The participant is invited to judge the behaviour of the main character in the dilemma and, on a scale of 1 (I completely reject) to 4 (I fully accept), expresses an opinion on each point (based on the performance of the main character). Through the results it is possible to determine the total C-score, the score for each dilemma and the score of the relationship between the different dilemmas. For the application of the questionnaire in Portugal, authorization was requested from Professor Bataglia, who is responsible for validation of the questionnaire to the Portuguese language and any necessary amendments were made with the author.

### Statistical Methods

The moral competence scores were described with the mean and SD (standard deviation), and the scores were compared in the two moments in which they were performed; this was conducted with *t* tests of paired samples. A multivariate linear regression analysis was performed for each score, with the difference between the two moments acting as dependent variables, and gender, age, and course as independent variables. A significance level of 5% was used. Frequency of student’s response, with differences in total scores up to five points or above, was evaluated between the two moments to determine the percentage of students with regression, stagnation, or increased moral competence.

### Ethical Aspects

Authorization for use of the questionnaire was granted by the Ethics Committees of the various schools in which the questionnaires were applied. Students were informed of the study objectives and gave their written consent voluntarily. Each questionnaire was assigned a code, chosen by the student, and they were asked to keep this code and insert it again for the second moment: the code did not allow the researchers to identify the student, as they did not have access to the student’s identification. The code only allowed for pairing the two questionnaires and determining if the student answered in the two moments.

## Findings

With a total of 918 students enrolled in the ethics course in different schools, 405 (44%) questionnaires were completed for both moments, of which 297 questionnaires were answered by nursing students and 108 by medical students. From those answered, one nursing student questionnaire and four medical student questionnaires were removed for 0-value responses in arguments for and against the main character’s action in the dilemma, rendering the questionnaires to be unfeasible. The total sample comprised 400 students with completed questionnaires, belonging to 296 nursing students and 104 medical students. In some schools, only a small number of students were in the first and last lessons (corresponding to the moment of application of the questionnaires), and had an extremely low response rate (7.5%, 13%, and 30%). Thus, to avoid bias, only the results from schools with a response rate above 50% (56%, 64%, and 90%) were analysed; nevertheless, results of the total sample can be found in the “Appendix”. Student samples with a response rate over 50% included 263 nursing students and 70 medical students, for a total of 333 students.

The sample characterization of schools with a response rate above 50% is presented in Table 1.

**Table 1:** Sample characterization

	Nursing students n = 263	Medical students n = 70
*Sex n (%)*		
Female	230 (88)	44 (63)
Male	33 (12)	26 (37)
Age median (min–max)	19 (18–44)	21 (20–39)

The sample of the two courses consists mainly of female students. Average age of nursing students was 19 and that of medical students was 21 years; the difference in ages can be explained by the fact that the nursing students attended the first and second year of the course and the medical students attended the fourth year.

The performance of the MCTxt dilemmas (C-worker, C-doctor, and C-judge), the moral competence total(C-score) and the relationship between dilemmas for nursing and medical students are shown in Table 2.

**Table 2:** Mean and SD of scores for nursing and medical students at both moments

	Nursing students n = 263			Medical students n = 70		
	First moment Mean (SD)	Second moment Mean (SD)	*p*	First moment Mean (SD)	Second moment Mean (SD)	*p*
*Score*						
Total	21.0 (13)	19.5 (13)	**0.046**	23.2 (16)	22.0 (16)	0.358
Worker	44.9 (23)	43.4 (23)	0.345	46.1 (22)	39.5 (22)	**0.033**
Doctor	34.4 (23)	30.0 (22)	**0.004**	33.4 (23)	32.2 (24)	0.704
Judge	43.0 (24)	43.3 (24)	0.819	44.3 (26)	45.2 (25)	0.771
*Relationship between*						
Worker and doctor	25.7 (14)	22.4 (14)	**0.001**	26.0 (17)	22.8 (16)	0.140
Worker and judge	27.4 (16)	27.4 (15)	0.996	30.7 (16)	29.3 (16)	0.496
Doctor and judge	24.0 (17)	22.5 (16)	0.125	25.7 (20)	24.7 (19)	0.674

Bold values indicate statistical significance in the results

Results show that students presented lower C-scores at the second moment of the questionnaire’s application. Nursing students showed a mean of the total score in the first moment of 21 points and in the second moment 19.5 points. Regarding medical students, the mean of the total score at the first moment was 23.2 points and in the second moment was 22 points, therefore less than 5 points between the two moments, which reveals, in average, a stagnation in students’ moral competence. Analysing the frequency of students’ responses, with some changes in the difference between the two moments, it was verified that approximately 29% of nursing students and 26% of medical students presented a difference of less than 5 points corresponding to a moral stagnation, as 39% of students in both courses came down 5 or more points revealing a moral regression and 32% of nursing students and 35% of medical students rose 5 or more points corresponding to an increase in moral competence.

Performance for each moral dilemma in the questionnaire was verified for all students, and there was a decrease in the worker’s and doctor’s score; this decrease was significant for nursing students in terms of the doctor’s dilemma. For medical students, the decrease was significant in relation to the worker’s dilemma. Regarding the judge’s score, there was an increase in the score for all students, although it showed no statistical significance. In terms of the relationship score between different dilemmas, there was a decrease in the second moment, though it was only significant in the worker and doctor relationship for nursing students. For nursing students, it was verified that in the relation between worker and judge, the score remained equal in the second moment, revealing a very close association between the results for worker and judge dilemma, as showed in previous studies [4].

Analysing the mean difference between the two moments of the questionnaire’s application, as shown in Table 3, there is a significant decrease in the total score. In a multivariate analysis, this decline does not seem related to gender (β = 1.623, *p* = 0.415), course (β = 1.111, *p* = 0.566), or age (β = 0.272, *p* = 0.269). There is also a significant decrease in relation to the doctor dilemma score, but no significant relationship was found between this decrease and gender (β = −0.722, *p* = 0.844), course (β = −2.718, *p* = 0.445), or age (β = 0.323, *p* = 0.475). Regarding the score of the relation between worker and doctor, there is also a significant decrease, with no connection found between this decrease and gender (β = 0.373, *p* = 0.881), course (β = 0.863, *p* = 0.721), or age (0.400, *p* = 0.194).

**Table 3:** Mean and respective confidence interval of 95% (CI 95%) for the 2nd moment/1st moment difference

	Mean difference	(CI 95%)
*Score*		
Total	− **1.61**	**(**− **3.04;** − **0.17)**
Worker	− 2.58	(− 5.35; 0.19)
Doctor	− **3.71**	**(**− **6.35;** − **1.06)**
Judge	0.47	(− 2.23; 3.18)
*Difference between*		
Worker and doctor	− **3.27**	**(**− **5.07;** − **1.47)**
Worker and judge	− 0.28	(− 2.00; 1.43)
Doctor and judge	− 1.40	(− 3.20; 0.39)

Bold values indicate statistical significance in the results

This significant changes in scores suggest that the decrease in students’ moral competence (total score, doctor score, relationship between worker and doctor) does not seem to be related to either gender, age or course.

## Discussion

Results obtained through this study demonstrate, on average, a decrease from the first to the second moment in the score of the moral competence of nursing and medical students, which may indicate that the teaching programmes may have had some success, but that there are gaps that do not allow most students to show an increase in scores of moral competence. As a way to fill these gaps, several authors propose measures such as curricular revision, the promotion of reflection and decision-making, and the use of methods that promote ethical argumentation and discussion of dilemmas, witnessed in clinical practice under the guidance of teachers and instructors with ethical training [2, 3, 8, 12, 20, 22].

We observe that for the nursing students present, in both moments, C-scores were lower to those of medical students, 2.2 points in the first moment and 2.5 points in the second moment, even though these scores do not correspond to a significant difference between groups, according to Lind [14], so we consider that in both groups it is necessary to implement teaching methods that promote moral competence. The motivation to act morally is associated with the system of values that each one builds in their life. Since values are related with affective dimension of moral behaviour it will be important to promote teaching methods that stimulate not only the cognitive domain but also the affective dimension of moral behaviour, such as visualization of films or playing games with colleagues [21]. The clinical practice also seems to be a factor that promotes moral development [2, 8, 20] and decision making [1], since the contact with the illness and the real ethical dilemmas may foster the cognitive and affective domain of behaviour. Is in the relationship between the concrete details of a particular case and the general principles that fosters a morally right action [1]. In our study none of the students had already experience in clinical setting which may be associated with the stagnation or regression in moral competence.

Regarding the C-scores obtained, we observe that nursing students in the first moment and medical students in the two moments present C-scores from 20.0 to 29.9 points, which correspond to a sufficient moral competence to choose an option in many dilemma situations, such that the requirement is not too high [16]. Regarding scores obtained by nursing students in the second moment, with a C-score of 19.5 points, this corresponds to a low moral competence (from 10.0 to 19.9) which demonstrates that the group has less moral competence than that needed to choose an option in a dilemma situation [16].

We verified that the nursing students in our sample obtained total scores, at the first moment of 21 points, higher than those obtained by Oliveira [19], with an initial total score of 17.6 points. Regarding medical students in our study, they presented initial total scores of 23.2 points, which are higher than those obtained, in a longitudinal study with medical students, by Serodio [21], including an initial total score of 19 points for the control group, that have conventional ethics classes, in which the expository method was mainly used; and 20.4 points for the group using complementary teaching with the KMDD. In Serodio study [21], it was verified that students presented lower scores of moral competence in the second moment of the questionnaires’ application for the control group, and higher scores for the group subject to KMDD, however in both cases corresponding to a moral stagnation [21]. These results reinforce to the need to use teaching methods based on the discussion of ethical dilemmas, which stimulate the development of reflection and critical judgment in students [6, 21, 22].

Several authors [7, 12, 18, 22, 23] found that medical students had higher moral competence at the beginning of the course than in the following years. In our study, the temporal distance between the two moments was shorter, from the beginning to the end of the same semester, but we found in the same way a decrease in moral competence scores.

Through data analysis, it was possible to verify that both nursing and medical students presented scores in the worker’s and judge’s dilemma, which differ from the doctor’s dilemma score. In both groups, the difference between the dilemmas of the worker and judge, in contrast to the dilemma of the doctor, is greater than 5 points, which according to Lind [15], indicates there is a phenomenon called moral segmentation. The phenomenon of moral segmentation was also observed in several studies [3, 17, 18], in which they similarly found disparate results for the two dilemmas of the MCT original version and for Oliveira [19], who applied the MCTxt questionnaire that included three moral dilemmas in Brazil. The doctor dilemma being related to the value of life and the practice of euthanasia, makes the task of making a decision for or against the main character’s attitude more difficult, givens the influence of social agents, such as religion or family environment, in decision-making. [4]. Each person, as a member of a community, learn early that there are moral responsibilities and rules to abide by, that are an integral part of a common moral, and this rules and principles end up influencing the moral action [5]. These results lead us to consider that there are more similarities between the results obtained in Portugal and Brazil, than with other countries of Europe where religion does not take on such a determining role. Besides that, there is a historical link between these countries, as Brazil was a former Portuguese colony, and both countries share language and cultural connections [18].

Interestingly, in the results of the relation between worker and judge for nursing students, the scores remained the same, revealing a very close association between these two dilemmas, as verified by Bataglia [4], whereas in the relationship between worker and doctor, there was a significant decrease, which may be related to moral segmentation. In relation to medical students, they presented close scores in the relationship between the worker and judge, in both moments of the questionnaire, while in the relationship between the worker and doctor, they presented a more pronounced difference, though not statistically significant.

## Limitations

One of the limitations were the schools that were excluded from analysis (they had a very low response rate, due to the organizational characteristics of the school and the ethics curricular unit). However, when analysing results of the total sample (“Appendix”), we verified that without exclusion of schools with a very low response rate, they were identical to the analysed schools, (a response rate above 50%), blurring the differences, as they were small and some were no longer statistically significant. We suspect this happens, because in schools with a low response rate, only those students who are extremely interested in the subjects end up being interviewed, which may have created a bias. These students, who are ostensibly interested in ethics, tended to decrease less the moral competence score, between the beginning and after the ethics curricular unit be taught.

The second moment of application of the questionnaire coincides with when students were subject to more evaluation moments and thus may have been tired, influencing their availability and attention in filling out the questionnaire in the second moment.

Another limitation of the study is that the questionnaire was validated for the Portuguese language, but the validation was made in Brazil. Although no formal cultural adaptation of the questionnaire was made, the author of the Portuguese language version collaborated on the adaptation of the questionnaire to be applied in Portugal.

Finally, as the ethics curricular unit is mandatory, it was not possible to form a control group in which this curricular unit had not been administered, as well as to verify if the differences between the two moments were similar without teaching ethics. Thus, the differences between the two moments cannot be attributed directly to the teaching that was administered between the two moments, since they may also be the result of a greater reflection of the dilemmas by the students. In fact, the first time the questionnaire was applied, students had no contact with these dilemmas, and in the second moment they already knew the dilemmas and had the opportunity to reflect on them and discuss them with third parties outside of the ethics class.

## Conclusion

The results obtained through our study revealed, that most of nursing and medical students present a stagnation or regression in the moral competence after being exposed to ethics education. These results may indicate that more attention is paid to the technical preparation of medical and nursing students, placing critical judgment and decision-making in the background. Given a rising number of ethical dilemmas, it is urgent to find ways to prepare health professionals to treat the patient as a whole, in all dimensions, having the patient´s well- being and quality of life as the ultimate goal, plus promoting the humanization of health care. Although the temporal distance between the two moments of the questionnaire’s application is only 4 months, this prompts us to consider what measures to implement to promote the development of moral competence in nursing and medical students. We present, as a possible solution to this problem, the revision of school curricula and the need for more training in ethics, using methods that stimulate a student’s interest, as the presentation and discussion of case studies, the development of a good learning environment were the students feel confident to demonstrate their feelings and opinions developing their critical and reflective thinking. We also consider that the contact with the patients and the discussion of real ethical dilemmas about clinical practice may help medical and nursing students to develop their ability to make decisions and act according to them. Future research related to the ethics teaching and the development of medical and nursing students’ moral competence should be conducted with attention to ethics curricula, and should include further studies comparing different teaching methods and their impact on students’ moral competence and also the role of clinical practice on students’ moral competence.

## Appendix

See Table 4.

**Table 4:** Mean and SD of scores in medical and nursing students—total sample

	Nursing n = 296			Medicine n = 104		
	First moment Mean (SD)	Second moment Mean (SD)	*p*	First moment Mean (SD)	Second moment Mean (SD)	*p*
*Score*						
Total	20.8 (13)	20.1 (14)	0.394	23.5 (15)	22.1 (15)	0.327
Worker	45.0 (23)	44.2 (23)	0.574	46.8 (22)	41.1 (22)	**0.019**
Doctor	34.3 (23)	30.9 (22)	**0.019**	33.5 (23)	34.1 (23)	0.799
Judge	42.4 (24)	43.8 (25)	0.398	47.2 (25)	48.4 (25)	0.631
*Difference between*						
Worker and doctor	25.5 (14)	23.1 (15)	**0.012**	26.0 (17)	23.5 (16)	0.122
Worker and judge	27.2 (16)	28.0 (16)	0.404	31.6 (16)	30.5 (16)	0.456
Doctor and judge	23.6 (17)	23.2 (17)	0.719	26.7 (20)	26.7 (19)	0.981

Bold values indicate statistical significance in the results
